# Doxycycline Induces Apoptosis of *Brucella Suis* S2 Strain-Infected HMC3 Microglial Cells by Activating Calreticulin-Dependent JNK/p53 Signaling Pathway

**DOI:** 10.3389/fcimb.2021.640847

**Published:** 2021-04-28

**Authors:** Zhao Wang, Yanbai Wang, Huan Yang, Jiayu Guo, Zhenhai Wang

**Affiliations:** ^1^ School of Clinical Medicine, Ningxia Medical University, Yinchuan, China; ^2^ Cerebrospinal Fluid Laboratory, The General Hospital of Ningxia Medical University, Yinchuan, China; ^3^ Emergency Department, The General Hospital of Ningxia Medical University, Yinchuan, China; ^4^ Neurology Center, The General Hospital of Ningxia Medical University, Yinchuan, China; ^5^ Diagnosis and Treatment Engineering Technology Research Center of Nervous System Diseases of Ningxia Hui Autonomous Region, The General Hospital of Ningxia Medical University, Yinchuan, China

**Keywords:** neurobrucellosis, *Brucella suis* S2 strain, human microglia, doxycycline, apoptosis, JNK/p53 signaling pathway

## Abstract

Neurobrucellosis is a chronic complication of human brucellosis that is caused by the presence of *Brucella* spp in the central nervous system (CNS) and the inflammation play a key role on the pathogenesis. Doxycycline (Dox) is a widely used antibiotic that induces apoptosis of bacteria-infected cells. However, the mechanisms of *Brucella* inhibition of microglial apoptosis and Dox induction of apoptosis are still poorly understood. In this study, we found that *Brucella suis* S2 strain (*B. suis* S2) increased calreticulin (CALR) protein levels and inhbited HMC3 cell apoptosis. Hence, we constructed two HMC3 cell line variants, one with stable overexpression (HMC3-CALR) and one with low expression of CALR (HMC3-sh-CALR). CALR was found to decrease levels of p-JNK and p-p53 proteins, as well as suppress apoptosis in HMC3 cells. These findings suggest that CALR suppresses apoptosis by inhibiting the JNK/p53 signaling pathway. Next, we treated HMC3, HMC3-CALR and HMC3-sh-CALR cell lines with *B. suis* S2 or Dox. Our results demonstrate that *B. suis* S2 restrains the JNK/p53 signaling pathway to inhibit HMC3 cell apoptosis *via* increasing CALR protein expression, while Dox plays the opposite role. Finally, we treated *B. suis* S2-infected HMC3 cells with Dox. Our results confirm that Dox induces JNK/p53-dependent apoptosis in *B. suis* S2-infected HMC3 cells through inhibition of CALR protein expression. Taken together, these results reveal that CALR and the JNK/p53 signaling pathway may serve as novel therapeutic targets for treatment of neurobrucellosis.

## Introduction

Neurobrucellosis is a chronic infectious disease and the molecular pathogenesis of this disease could have occurred as a result of *Brucella* infection of microglia and astrocytes, which promotes the secretion of pro-inflammatory mediators to damage neurons (García Samartino et al., 2010; Rodríguez et al., 2017; Rodríguez et al., 2019). Therefore, it is an infrequent but life-threatening complication of brucellosis (Raina et al., 2016). The atypical clinical manifestations of neurobrucellosis can lead to underdiagnosis and some patients develop a chronic infection (Zhao et al., 2016) that is probably caused by *Brucella* infection and replication within microglia, astrocytes and brain endothelial cells (García Samartino et al., 2010; Marim et al., 2017; Miraglia et al., 2018). Microglia are a kind of unique myeloid cells in the central nervous system (CNS) (Ginhoux et al., 2010; Butovsky et al., 2014; Gosselin et al., 2017; Sousa et al., 2017) that play an important role in host defense against bacterial infections of the brain (Nau et al., 2014). Upon entry into host cells, *Brucella* enters the endosomes *via* type IV secretion system (T4SS) or BvrR/BvrS system to form the *Brucella*-containing vacuoles (BCVs), which helps *Brucella* to colonize in the endoplasmic reticulum (ER) to ensure its survival, replication and egress (Ahmed et al., 2016; Głowacka et al., 2018; Celli, 2019). The intracellular replication of *Brucella* assists bacterial evasion of the host innate or adaptive immune system, and inhibition of apoptosis is a strategy of *Brucella* to evade the immune response to establish chronic infection (Ahmed et al., 2016). However, the mechanisms by which *Brucella* suppresses apoptosis are poorly understood.

Calreticulin (CALR) is a calcium storage protein in the endoplasmic reticulum (ER) (Yang Y. Z. et al., 2019). It has been reported that the expression of CALR protein is increased in tumor cells, which is associated to increased cell death in different tumours (Sun et al., 2017). Additional report indicates that the CALR plays a role in antiapoptosis and antigen presentation within host cell (Xu et al., 2017). For example, the increased expression of CALR protein often results in decreased rates of neuronal apoptosis after cerebral ischemia (Chen et al., 2015). We argue that the effect of CALR on the induction or inhibition of apoptosis may be associated with the variability in the proliferation and metabolism of cancerous and neuronal cells. The C-JUN N-terminal kinase (JNK) signaling pathway is a critical mediator of ER-related apoptosis (Maeyashiki et al., 2020); ER stress triggered by bacteria (Fung and Liu, 2017) can activate apoptosis signal-regulating kinase 1 (ASK1) and transduce signals to mitogen-activated protein kinase kinase 4 (MKK4), which synergistically phosphorylates and activates JNK (Rusnak et al., 2018). Once activated, phosphorylated JNK translocates into the nucleus and phosphorylates p53 (Lien et al., 2015) to induce apoptosis of the host cells (Lim et al., 2020). Nevertheless, the association between CALR and the JNK signaling pathway in the regulation of apoptosis has not received research attention.

*Brucella* inhibits apoptotic mechanism in infected macrophages, which favors intracellular growth of the bacteria (Ahmed et al., 2016). This indicates that apoptosis inhibition is a strategy of *Brucella* for intracellular replication that in turn helps the bacteria to escape the attack of the host immune system. Therefore, in theory, bacterial survival can be limited by apoptosis of infected cells (Guo et al., 2016). Doxycycline (Dox), a tetracycline antibiotic used in treating bacterial infections (Nakagawa et al., 2018), has been reported to induce apoptosis by triggering ER stress (Matsumoto et al., 2017). Importantly, however, the mechanism underlying Dox-induced apoptosis remains unknown.

Building on the above data, we used human microglia clone 3 (HMC3) cell line, which was developed in the laboratory of Prof Marc Tardieu in 1995 (Janabi et al., 1995) and has been used in different laboratories under different denominations (Dello Russo et al., 2018). We then constructed HMC3 cell lines in which CALR protein is over-expressed (HMC3-CALR) or down-regulated (HMC3-sh-CALR) to investigate the effect of CALR on apoptosis and JNK signaling in HMC3 cells. Next, we examined the impact of *Brucella suis* S2 strain (*B. suis* S2, attenuated strain) and Dox on CALR protein expression and JNK/p53-dependent apoptosis in HMC3 cells. Finally, we investigated the mechanism of Dox-induced apoptosis in *B. suis* S2-infected HMC3 cells.

## Materials and Methods

### Antibodies and Lentivirus

We obtained primary antibodies against Calreticulin (#GR3252549-6), GAPDH (#GR200347-16), ASK1 (#GR3212614-6), MKK4 (#GR297166-6), p-MKK4 (Ser80, #GR24626-12), and p-JNK (Thr183+Tyr185, #GR3268514-1) from Abcam (Cambridge, UK) and primary antibodies against JNK (#I09181295), p-JNK (#I09261813), p53 (#I09171919), and p-p53 (#I11042504) from Wanlei Biotechnology (Shenyang, CHN). The primary antibody against β-actin (#AH11286487) was provided by Bioss Biotechnology (Beijing, CHN). Hanbio Biotechnology (Shanghai, CHN) provided CALR- NC-ZsGreen lentivirus, CALR-ZsGreen lentivirus, sh-NC-ZsGreen lentivirus and sh-CALR-ZsGreen lentivirus.

### Bacteria

*B. suis* S2 was provided by Ningxia Key Laboratory of Clinical Pathogenic Microorganisms (Yinchuan, CHN) and grown on soybean casein digest agar media (Oingdao Hope Biotechnology, Qingdao, CHN) at 35°C in an incubator with 5% CO_2_. After 3 days of growth on agar, single colonies of *B. suis* S2 were transferred to 10 ml tryptic soy broth (Oingdao Hope Biotechnology, Qingdao, CHN) at 35°C in a thermostat shaker (200 rpm) for 36 h. Bacteria were collected by centrifugation at 4500 rpm for 5 min and washed twice with 5 ml precooled phosphate buffered saline (PBS; Hyclone, Shanghai, CHN). *B. suis* S2 density was determined by turbidimetry. Bacterial culture was carried out at the Ningxia Key Laboratory of Clinical Pathogenic Microorganisms, a biosafety level PII laboratory.

### Cell Culture

HMC3 cells were purchased from Kelei Biotechnology (Shanghai, CHN) and authenticated by STR analysis (Biowing Applied Biotechnology, Shanghai, CHN). HMC3 cells were cultured in high glucose Dulbecco’s modified Eagle medium (DMEM; Hyclone, Shanghai, CHN) supplemented with 1% penicillin-streptomycin (Solarbio Life Sciences, Beijing, CHN) and 15% fetal bovine serum (FBS; GIBCO, Grand Island, USA) at 37°C with 5% CO_2_. When HMC3 cells reached 75% confluency in 10 cm dishes, cells were washed with prechilled PBS, diluted in a 1:2 ratio, and transferred to new petri dishes.

### Cell Transfection and The Generation of Cell Lines With Stable Expression

HMC3 cells subcultured for three passages were plated onto a 96-well plate (Corning Incorporated, Corning, USA) containing 1 × 10^4^ cells in 100 μl complete medium per well. Lentiviral vectors Lv-CALR-NC-ZsGreen-PURO and Lv-sh-NC-ZsGreen-PURO were used as controls. HMC3 cells in 96-well plate were inoculated with Lv-CALR-NC-ZsGreen-PURO at multiplicities of infection (MOI) of 5, 10, 20, 40 and 80. ZsGreen protein expression was detected by fluorescence microscopy (Olympus, Tokyo, Japan) after 72 h. In subsequent experiments, HMC3 cells were transduced with lentivirus at an MOI of 40, which ensured the high lentiviral transfection efficiency with minimum damage. Each of the four lentiviruses (MOI=40) Lv-CALR-NC-ZsGreen, Lv-CALR-ZsGreen, Lv-sh-NC-ZsGreen and Lv-sh-CALR-ZsGreen were added to cultured cells, in FBS-free medium. After 24 h of lentivirus transfection at 37°C, the virus-containing medium was changed to complete medium and transfected HMC3 cells were incubated for 48 h under the same conditions. Next, stably transfected HMC3 cell lines were selected by puromycin (5 μg/ml) until the non-transfected HMC3 cells died off; depending on virus inoculum, the 4 resulting transfected cell lines were named, respectively, HMC3-CALR-NC, HMC3-CALR, HMC3-sh-NC, HMC3-sh-CALR.

### Infection in Vitro

HMC3 cells were plated at a density of 2×10^6^ cells per dish in 100 mm-diameter dishes in antibiotic-free medium. When HMC3 cells reached 75% confluency, one batch of cells was infected with *B. suis* S2 (MOI = 50) (Yang J. et al., 2019) for 1, 2, 4 and 8 h and another batch exposed to the same bacteria at MOI of 25, 50, 100 and 200 for 2 h. Immediately afterwards, HMC3 cells were washed several times with pre-chilled PBS in order to thoroughly remove non-internalized *B. suis* S2, then harvested for subsequent assays.

### Doxycycline Treatment in Vitro

Doxycycline (Dox; MedChem Express, Monmouth Junction, USA) was dissolved in PBS at a concentration of 10 mM and stored at -80˚C. The storage solution was diluted to final concentration with PBS when needed. When the HMC3 cells reached 75% coverage, they were treated with 40 μM Dox (Matsumoto et al., 2017) for 6, 12 and 24 h. Another batch of HMC3 cells was exposed to Dox at various concentrations (20, 40, 80 and 160 µM) for 12 h (Alexander-Savino et al., 2016). Additionally, HMC3, HMC3-CALR and HMC3-sh-CALR cell lines were each treated with 160 µM Dox for 12 h, and the same concentration of Dox was also used to treat *B. suis* S2-infected HMC3 cells for 12 h.

### Cell Counting Kit-8 (CCK-8) Assay

HMC3 cells were plated at a density of 1 × 10^4^ cells per well onto 96-microtiter plates and grown at 37°C in 5% CO_2_. After 12 h, HMC3 cells were treated with Dox at the concentrations and treatment periods above; each group had six parallel samples. Immediately afterwards, 10 µl CCK-8 reductant (Meilunbio, Dalian, CHN) was dripped into each well under light-protected conditions and cells were continuously incubated for 2 h at 37°C in 5% CO_2_. Optical density (OD) was measured at 450nm with a microplate reader (BioTek, Biotek Winooski, USA). Cell viability was defined as survival rate (%) = (OD value of treatment group/OD value of non-treated group) × 100%.

### Flow Cytometry Analysis With Annexin V-FITC/PI

Apoptotic HMC3 cells were stained with the BBcellProbe™ Annexin V-FITC/PI double-stained kit (BestBio, Shanghai, CHN). Treated HMC3 cells were digested by prewarming trypsin at 37°C for 5 min and harvested *via* centrifugation (1000 rpm, 5 min) at 4 degrees. After two rinses in 1 ml prechilled PBS, HMC3 cells were resuspended in 400 µl of Annexin V binding buffer to give a density of 1 × 10^6^ cells per ml. Next, 5 µl of Annexin V-FITC staining solution was added to the cell suspension, which was incubated for 15 min at 4 degrees in dark. Next, 10 µl of propidium iodide (PI) staining solution was added to the cell suspension. After a 5 min incubation, the apoptotic rate of the HMC3 cells (about 4 × 10^5^ cells in each group) was analyzed and calculated with a flow cytometer (BD Biosciences, San Jose, USA). Control HMC3 cells were used to apply fluorescence compensation to the data and cells were gated on the control HMC3 cells. FlowJo V10 was used for analysis of results. The four quadrants of rectangular gate—Q1, Q2, Q3 and Q4 represent necrotic, late apoptotic, early apoptotic and live cells respectively. The total cell apoptotic percentage includes both early and late apoptotic rates (Q3 + Q2).

### Flow Cytometry Analysis With Annexin V-APC/7-AAD

To prevent artifacts caused by emission of ZsGreen protein fluorescence on detection results, Annexin V-APC and the 7-AAD Apoptosis Kit (US Everbright, Suzhou, CHN) were used to evaluate apoptotic cells in the HMC3-CALR and HMC3-sh-CALR cell lines. Cell suspensions were prepared as described above. 5 µl of Annexin V-APC and 10 µl of 7-AAD staining solution were added to 100 µl of each cell suspension and the mixture was incubated at ambient temperature for 15 min in the dark. Finally, 400 µl of Annexin V binding buffer was added to the cell mixtures (about 4 × 10^5^ cells in each group) and cells were detected with a flow cytometer. Cells were gated on the control HMC3 cells, which were used to apply fluorescence compensation to the data. And data were analyzed by FlowJo V10 software. The different quadrants Q1, Q2, Q3 and Q4 represent necrotic cells, late apoptotic cells, early apoptotic cells and viable cells respectively. The sum of quadrants Q3 and Q2 was used to calculate the apoptosis ratio.

### Western Blot Analysis

HMC3 cells were plated at a density of 2 × 10^6^ cells per dish in 100 mm-diameter dishes. When cells reached 90% confluency, the expression levels of CALR, JNK pathway-related proteins, p53 and p-p53 in each cell line were assessed using western blots. A Whole-cell Protein Acquisition Kit (KeyGEN Biotech, Nanjing, CHN) was used to extract total proteins from each cell line; these extracts were centrifuged at 4°C for 50 min at 13,500 rpm. Total-protein concentrations were assayed using a BCA Protein Content Assay Kit (KeyGEN Biotech, Nanjing, CHN). The protein solutions (70 μg/lane) from each group were separated on 8-12% SDS-PAGE gels (KeyGEN Biotech, Nanjing, CHN) and electro-blotted onto 0.45 µm polyvinylidene difluoride (PVDF) membranes (Merck Millipore, Billerica, USA). Non-specific binding sites on the membranes were blocked at ambient temperature for 1.5 h with 5% (w/v) skimmed milk dissolved in Tris Buffered Saline-Tween 20 (TBST); the membranes were incubated with primary antibodies (1:500-1:1000) at 4˚C overnight. After rinsing with TBST, the membranes were incubated with IRDye^®^ 800CW Goat anti-Rabbit IgG (1:7000; LI-COR Biosciences, Lincoln, Nebraska, USA) at ambient temperature for 1 h. Then, the targeted proteins were assayed using Odyssey Clx Infrared Imaging System (LI-COR Biosciences, Lincoln, Nebraska, USA). Experiments were carried out three independent times. The mean optical densities (MOD) of the target protein was normalized with respect to β-actin or GAPDH and the level of each protein was represented by the relative MOD.

### RT-qPCR Assay

HMC3 cells and HMC3 cell lines solidly transfected with CALR-NC, CALR, sh-NC or sh-CALR expression plasmids were plated into 60 mm-diameter dishes. When cell confluency reached about 75%, total cellular RNA was isolated using RNAsimple Total RNA Kit (TIANGEN Biotech, Beijing, CHN) and cDNA in each group was synthesized according to manufacturer instructions of the Transcriptor First Strand cDNA Synthesis Kit (Thermo Fisher Scientific, Waltham, USA). The cDNA samples were stored at -80°C. Using a ChamQ SYBR qPCR Master Mix (Vazyme Biotech, Nanjing, CHN) and LightCycler^®^ 480 II Authorized Thermal Cycler (Roche, Basel, Switzerland), qPCR was carried out at 95°C for 30 s, 40 cycles of 95°C for 10 s, 56°C for 30 s and 72°C for 30 s; the conditions of the melting curve analysis were as follows: at 95°C for 15 s (DNA denaturation), at 60°C for 60 s (double stranded DNA) and at 95°C for 15 s (single stranded DNA). The melting temperature (Tm) of the amplicons was 84.5°C. The CALR and GAPDH primers used were designed and synthesized by Sangon Biotech (Shanghai, CHN). The sequences for the primers are as follows: *CALR*, CCAACGATGAGGCATACGCTGAG, and GCTCCTCGTCCTGTTTGTCCTTC; *GAPDH*, CAAGGTCATCCATGACAACTTTG, and GTCCACCACCCTGTTGCTGTAG. The primer specificity was confirmed by agarose gel electrophoresis and melting curve analysis. For RT-qPCR, four times independent biological replicates were performed and relative differences mRNA levels were analyzed using the 2^-ΔΔCT^ method.

### Immunofluorescence Staining

Cells were grown on poly-L-lysine-coated cover slips (NEST Biotechnology, Wuxi, CHN), immersed in paraformaldehyde (4%) for 10 min, permeabilized with TritonX-100 (0.3%) for 15 min, and blocked for 30 min with 5% common goat serum (Boster Biotech, Wuhan, CHN) at ambient temperature. Next, the prepared cell samples were incubated with p-JNK antibody (1:100) at 4˚C overnight. After rinsing twice with TBST, samples were incubated with Rhodamine (TRITC)-Goat anti-Rabbit IgG (H+L) (1:200; ZSGB-BIO, Beijing, CHN) at 37°C for 1 h. Nuclei were visualized with DAPI and cell samples were sealed with anti-fluorescence quencher. Finally, images were visualized with a FV 1000 laser confocal microscope (Olympus, Tokyo, Japan).

## Statistical Analysis

In this study, 3 independent biological replicates were performed for each experiment and the results indicated as means ± standard deviation (SD). GraphPad Prism 8.0.2 was used to analyze experimental data. These data were first checked for normality with the Shapiro-Wilk test and the homogeneity of variance was analyzed using a Brown-Forsythe test. One-way analysis of variance (ANOVA) was use to analyze intergroup differences; values of p ≤ 0.05 were considered statistically significant.

## Results

### Effects of the *B. suis* S2 on Apoptosis and Levels of CALR, p-JNK and p-p53 at Different Timepoints

**Figure 1:**
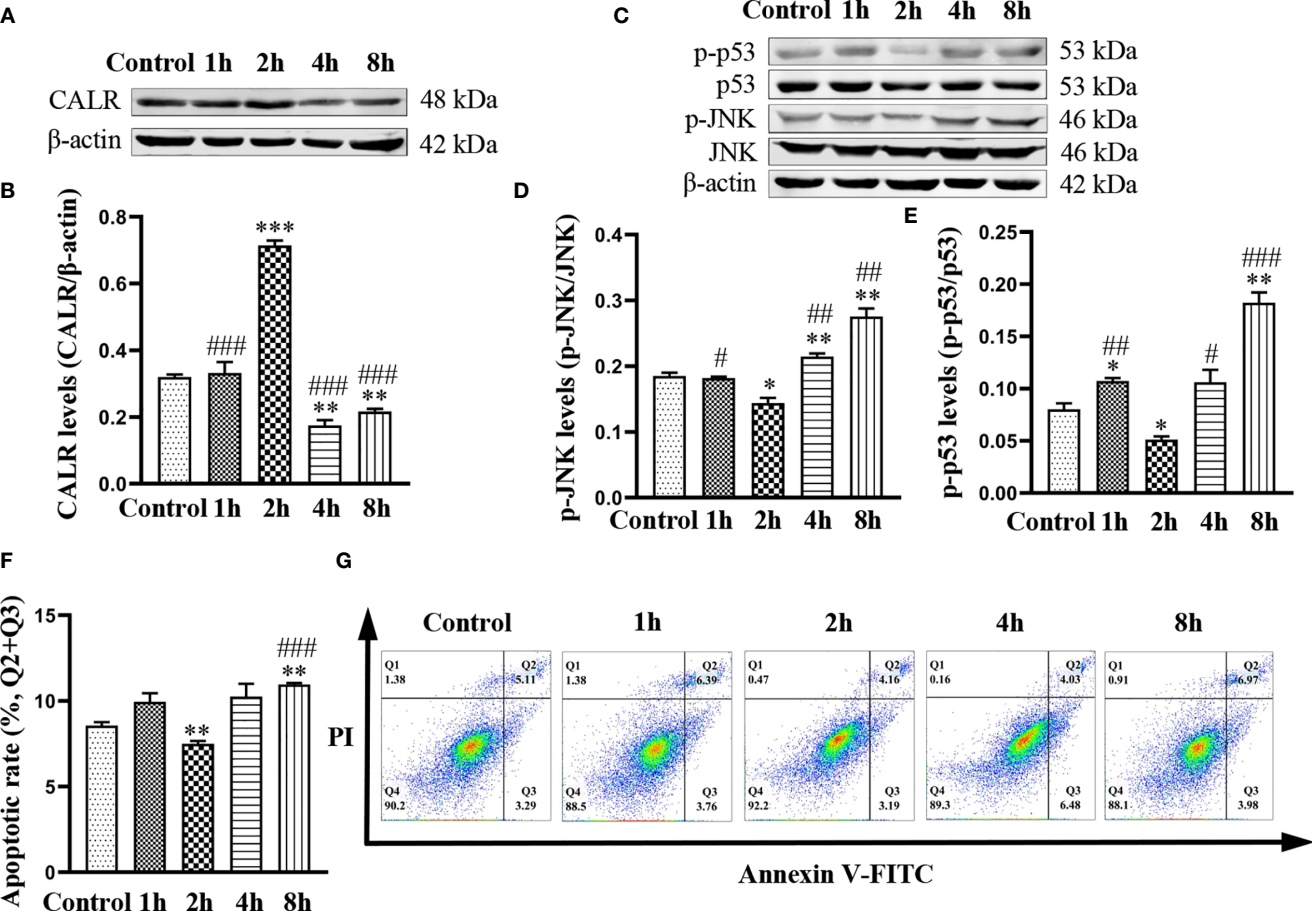
Effects of *B. suis* S2 on HMC3 cells at different timepoints. HMC3 cells were infected with *B. suis* S2 at a MOI of 50 for 1, 2, 4 and 8 h. **(A)** Western blots and **(B)** analysis of CALR protein in *B. suis* S2-infected HMC3 cells at different timepoints. **(C)** Western blot assay was used to measure protein levels of p-JNK and p-p53, and **(D, E)** the ratios of phosphorylated protein to total protein were calculated using densitometric analysis. **(G)** Plots of HMC3 cells apoptosis were generated using flow cytometry (Q1: necrotic cells, Q2: late apoptotic cells, Q3: early apoptotic cells, Q4: survival cells) and **(F)** analysis of apoptotic rates, the total apoptosis calculated as (Q2 + Q3). Data are presented as means ± SD; n = 3 independent repetitions; ^*^ *P* < 0.05, ^**^ *P* < 0.01, ^***^ *P* < 0.001 *versus* control; ^#^ *P* < 0.05, ^##^ *P* < 0.01, ^###^ *P* < 0.001 *versus* 2 h.

In order to investigate the effects of *B. suis* S2 on levels of CALR, p-JNK and p-p53, we infected HMC3 cells with bacteria at a MOI of 50 (Yang J. et al., 2019) for 1, 2, 4 and 8 h. Western blot results indicated that protein levels of CALR were the highest in the 2 h group, while the CALR protein levels in the 4 and 8 h groups were significantly reduced compared with those in the control group (**Figures 1A, B**). However, significantly lower levels of p-JNK and p-p53 proteins were expressed in the 2 h group compared with those in the other 4 groups. Compared with the control group, the p-JNK and p-p53 protein levels in the 8 h group were significantly increased (**Figures 1C–E**). Because *Brucella* has been reported to inhibit apoptosis in macrophages (Ahmed et al., 2016; Ma et al., 2020), we examined apoptosis in *B. suis* S2-infected HMC3 cells at different timepoints by flow cytometry (**Figure 1G**). Flow cytometric analysis indicated that compared with the control group, the apoptosis rate in HMC3 cells was reduced in the 2 h group but was increased in the 8 h group (**Figure 1F**). These results demonstrate that *B. suis* S2 increases protein levels of CALR, and decreases p-JNK and p-p53 protein levels and apoptosis in HMC3 cells after 2 h of infection at a MOI of 50. However, *B. suis* S2 can also decrease CALR protein levels, and increase p-JNK and p-p53 protein levels and apoptosis in HMC3 cells after 8 h of infection.

### *B. suis* S2 Influences Apoptosis and Levels of CALR, p-JNK and p-P53 at Different MOI Ratios

**Figure 2:**
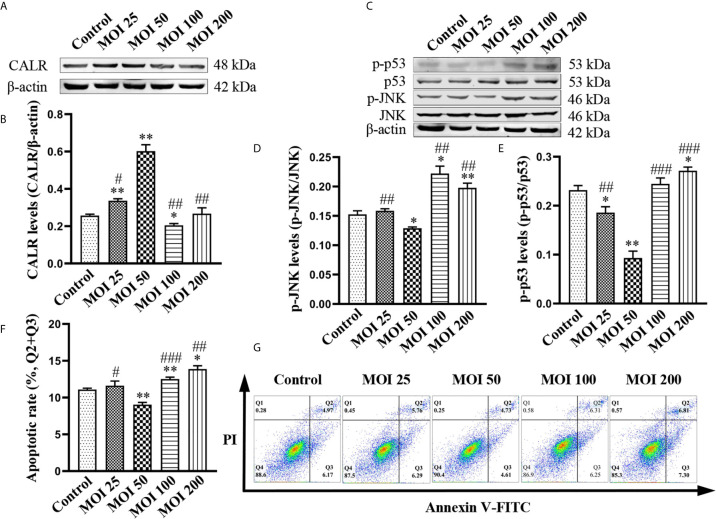
Effects of *B. suis* S2 on HMC3 cells at different MOI. HMC3 cells were exposed to *B. suis* S2 at MOI of 25, 50, 100 and 200 for 2 h. **(A)** Western blot analysis and **(B)** quantification of CALR protein expression in each group. **(C)** Protein levels of p-JNK and p-p53 were detected by western blotting and **(D, E)** the ratio of phosphorylated-to-total proteins was used to indicate quantification of phosphorylation proteins. **(G)** Apoptosis of HMC3 cells was determined with flow cytometry (Q1: necrotic cells, Q2: late apoptotic cells, Q3: early apoptotic cells, Q4: survival cells) and **(F)** the apoptosis rates were revealed as (Q2 + Q3). Data are indicated with means ± SD; n = 3 independent experiments; ^*^ *P* < 0.05, ^**^ *P* < 0.01 *versus* control; ^#^ *P* < 0.05, ^##^ *P* < 0.01, ^###^ *P* < 0.001 *versus* MOI 50.

To further determine the effect of *B. suis* S2 on inhibition of apoptosis in HMC3 cells, we exposed HMC3 cells to bacteria at MOI of 25, 50, 100 and 200 for 2 h. Results from western blotting revealed that CALR protein levels in the MOI 50 group were significantly higher than in the other groups (**Figures 2A, B**). The protein levels of p-JNK and p-p53 in the MOI 50 group were markedly lower than those of the other groups. However, compared with the control group, the protein levels of p-JNK and p-p53 in the MOI 200 group were significantly increased (**Figures 2C–E**). Flow cytometry analysis confirmed that apoptosis rate in the MOI 50 group was notably lower compared with the other 4 groups. But the rates of apoptosis were significantly increased in the MOI 100 and MOI 200 groups compared with the control group (**Figures 2F, G**). Collectively, these data indicate that *B. suis* S2 increases CALR protein levels, while reducing the levels of p-JNK and p-p53 after 2 h of infection at an MOI of 50. *B. suis* S2 also inhibits apoptosis of HMC3 cells in the same conditions. However, *B. suis* S2 can also increase p-JNK and p-p53 protein levels and apoptosis in HMC3 cells after 2 h of infection at an MOI of 200.

### CALR Suppresses Apoptosis *via* Decreasing the Levels of p-JNK and p-P53

**Figure 3:**
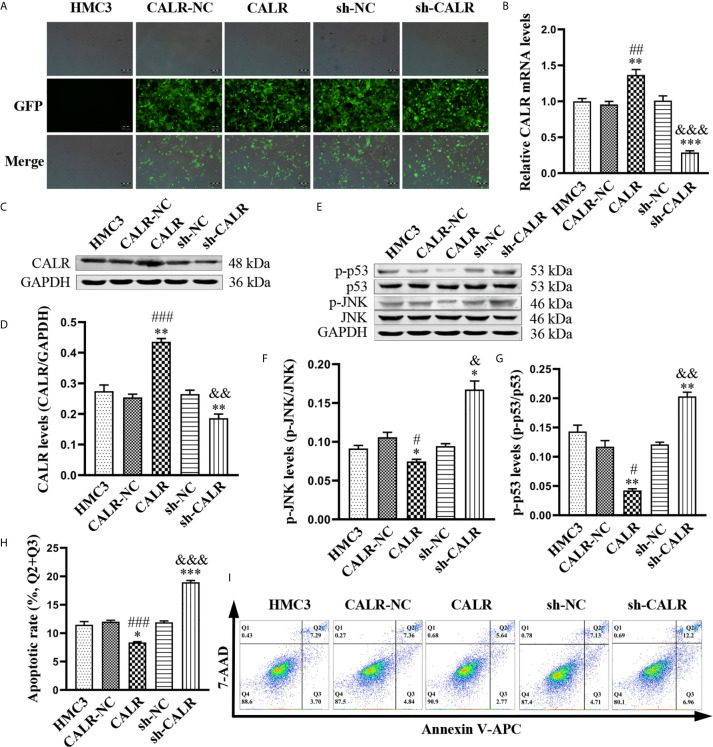
CALR inhibits p-JNK and p-p53, thus preventing apoptosis of HMC3 cells. **(A)** ZsGreen-expressing cells were observed with fluorescence microscopy after 72 h of lentiviral transfection. Scale bars = 200 µm. **(B)** CALR mRNA expression in each cell line was evaluated by RT-qPCR assay. **(C)** Protein expression of CALR was analyzed using western blot assay and **(D)** GAPDH was used as an internal reference protein to quantify CALR bands. **(E)** Protein levels of p-JNK and p-p53 were analyzed with western blot assay; **(F, G)** phosphorylated protein contents are indicated by specific phosphorylated-to-total-protein ratios. **(H)** Cell apoptosis was detected and **(I)** the apoptotic rates were calculated with flow cytometry (Q1: necrotic cells, Q2: late apoptotic cells, Q3: early apoptotic cells, Q4: survival cells), the total apoptosis calculated as (Q2 + Q3). Data are presented as means ± SD; n = 3 independent experiments; ^*^ *P* < 0.05, ^**^ *P* < 0.01, ^***^ *P* < 0.001 *versus* HMC3; ^#^ *P* < 0.05, ^##^ *P* < 0.01, ^###^ *P* < 0.001 *versus* CALR-NC; ^&^ *P* < 0.05, ^&&^ *P* < 0.01, ^&&&^ *P* < 0.001 *versus* sh-NC.

CALR has been reported to reduce cell apoptosis *via* inhibiting apoptosis-associated proteins (Xu et al., 2014); activated JNK catalyzes phosphorylation of p53, resulting in the induction of apoptosis (Dhanasekaran and Reddy, 2017). However, the relationship between CALR and p-JNK is unknown. Therefore, in order to investigate the effect of CALR on p-JNK, we used HMC3 cell lines expressing CALR-NC-ZsGreen, CALR-ZsGreen, sh-NC-ZsGreen and sh-CALR-ZsGreen (**Figure 3A**). RT-qPCR analysis and western blot assays indicated that both CALR mRNA (**Figure 3B**) and protein (**Figures 3C, D**) levels were significantly increased in the CALR group, when compared with the HMC3 and CALR-NC groups and that CALR mRNA (**Figure 3B**) and protein (**Figures 3C, D**) levels were markedly decreased in the sh-CALR group when compared with the HMC3 and sh-NC groups. Western blot assays of p-JNK and p-p53 in each group (**Figure 3E**) indicated that protein levels of p-JNK and p-p53 were significantly decreased in the CALR group compared with the HMC3 and CALR-NC groups (**Figures 3F, G**), but significantly elevated in the sh-CALR group compared with the HMC3 and sh-NC groups (**Figures 3F, G**). To examine the effect of CALR protein on apoptosis in HMC3 cells, we analyzed apoptotic cells *via* flow cytometry (**Figure 3I**). The results demonstrated that the apoptotic rate in the CALR group was decreased compared with that of the HMC3 and CALR-NC groups, and the apoptotic rate in the sh-CALR group was higher than that in the HMC3 and sh-NC groups (**Figure 3H**). Altogether, these data indicate that CALR protein downregulates p-JNK and p-p53 protein levels and consequently suppresses apoptosis in HMC3 cells.

### *B. suis* S2 Prevents JNK/p53-Dependent Apoptosis by Inducing Expression of CALR Protein

**Figure 4:**
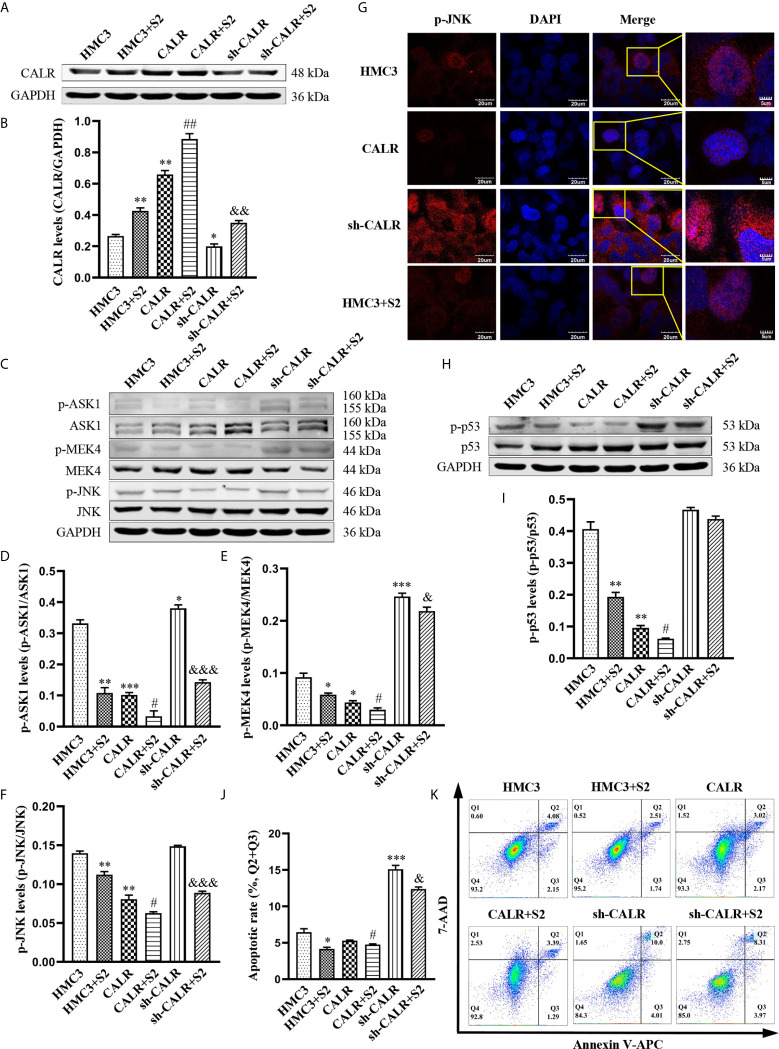
*B. suis* S2 induces CALR protein expression and further inhibits JNK/p53-dependent apoptosis. HMC3, HMC3-CALR and HMC3-sh-CALR cell lines were infected with *B. suis* S2 at a MOI of 50 for 2 h. **(A)** Western blot assay was used to determine CALR protein expression and **(B)** to quantify protein levels relative to GAPDH. **(C)** Protein levels of p-ASK1, p-MEK4 and p-JNK were measured with western blot assay and **(D–F)** protein phosphorylation was quantified with densitometry. **(G)** Nuclear localization of p-JNK protein was determined by immunofluorescence assay. Scale bars = 20 µm. **(H)** Protein levels of p-p53 were determined by western blot **(I)** assay and quantitative analysis of p-p53 protein. **(J, K)** Apoptosis rates of all groups were analyzed by flow cytometry (Q1: necrotic cells, Q2: late apoptotic cells, Q3: early apoptotic cells, Q4: survival cells), the total apoptosis calculated as (Q2 + Q3). Data are presented in means ± SD; All experiments were repeated 3 times independently; ^*^ *P* < 0.05, ^**^ *P* < 0.01, ^***^ *P* < 0.001 *versus* HMC3; ^#^ *P* < 0.05, ^##^ *P* < 0.01 *versus* CALR; ^&^ *P* < 0.05, ^&&&^ *P* < 0.001 *versus* sh-CALR.

To further investigate the regulatory effect of *B. suis* S2-induced CALR protein on the JNK/p53 signaling pathway, we infected HMC3, HMC3-CALR and HMC3-sh-CALR cell lines with *B. suis* S2. A western blot assay was used to determine the protein levels of CALR, p-ASK1, p-MEK4 and p-JNK (**Figures 4A, C**). The results suggested that protein expression of CALR in the HMC3+S2, CALR+S2 and sh-CALR+S2 groups was significantly increased over that of the corresponding controls, and that CALR protein levels were higher in the HMC3+S2 and CALR groups, and lower in the sh-CALR group, compared with the HMC3 group (**Figure 4B**). Nevertheless, the protein levels of p-ASK1, p-MEK4 and p-JNK were significantly decreased in the *B. suis* S2-infected groups relative to the corresponding control groups and the levels of these three proteins were reduced in HMC3+S2 and CALR groups compared to the HMC3 group (**Figures 4D–F**). However, the levels of p-ASK1 and p-MEK4 were higher in the sh-CALR group compared with the HMC3 group (**Figures 4D, E**). As is well known, phosphorylated JNK translocates to the nucleus and induces cell apoptosis by activating pro-apoptotic genes (Zeke et al., 2016). We detected nuclear localization of p-JNK protein *via* immunofluorescence techniques and observed that *B. suis* S2 did not significantly diminish p-JNK protein levels in the nucleus (**Figure 4G**). Western blot assay also showed that p-p53 protein levels were substantially decreased in the HMC3+S2 and CALR+S2 groups compared to the corresponding controls; compared with the HMC3 group, protein levels of p-p53 in the HMC3+S2 and CALR groups were also significantly reduced (**Figures 4H, I**). Flow cytometry results demonstrated that apoptotic rates were markedly reduced in the *B. suis* S2-infected groups compared to the corresponding control groups (**Figures 4J, K**). These results suggest that *B. suis* S2 inhibits the JNK/p53 signaling pathway and further prevents HMC3 cells apoptosis by promoting expression of CALR protein.

### Effects of Dox on CALR Protein Expression and Viability of HMC3 Cells

**Figure 5:**
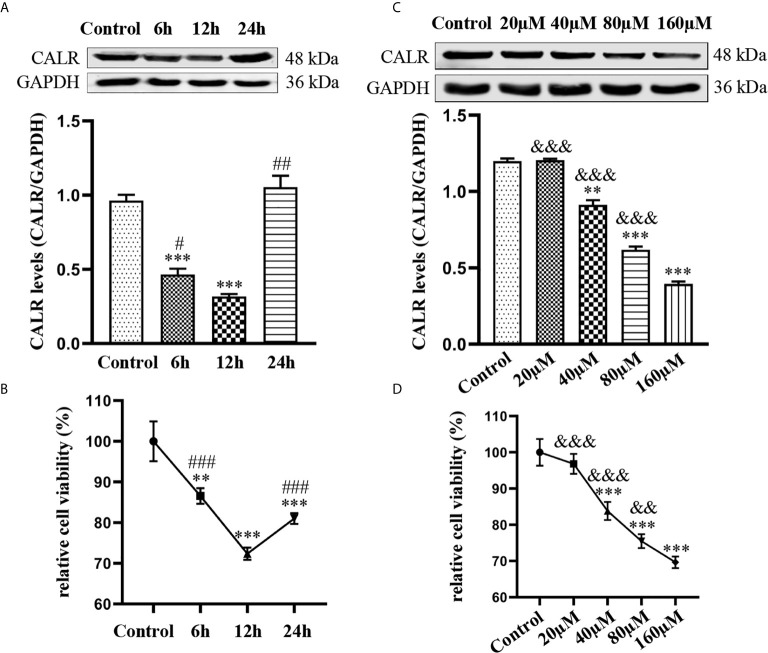
Effects of Dox on CALR protein expression and viability of HMC3 cells. **(A)** HMC3 cells were treated with 40µM Dox over different periods of time (0, 6, 12 and 24 h), and CALR protein expression was determined by western blot assay (mean ± SD, n = 3). **(B, D)** HMC3 cell viability was analyzed using a CCK-8 assay; results were calculated as survival rates relative to control (mean ± SD, n = 6). **(C)** HMC3 cells were treated with different concentrations of Dox (0, 20, 40, 80 and 160µM) for 12 h and CALR protein levels were measured using western blot assay (mean ± SD, n = 3). ^**^ *P* < 0.01, ^***^ *P* < 0.001 *versus* control; ^#^ *P* < 0.05, ^##^ *P* < 0.01 *versus* 12 h ^###^ *P* < 0.001; ^&&^ *P* < 0.01, ^&&&^ *P* < 0.001 *versus* 160µM.

Dox not only has antibacterial activity; it also can inhibit cell activity and further induce cell apoptosis (Melenotte and Raoult, 2018). To investigate the impact of Dox on CALR expression and HMC3 cell viability, we treated HMC3 cells with various concentrations of Dox over different periods of time. Expression of CALR protein was measured by western blot assay, which showed that CALR protein expression was lower in the 12 h group than in the other groups (**Figure 5A**). The results also suggested that the CALR levels were significantly decreased in the 160µM Dox-treatment group compared with the other 4 groups (**Figure 5C**). HMC3 cell viability was assessed using a CCK-8 assay, which indicated that treatment with 40µM Dox for 12 h reduced HMC3 cell viability more strongly than other treatments (**Figure 5B**), and that HMC3 cell viability was decreased in the 160µM group when compared with the remaining groups (**Figure 5D**). These data indicate that both CALR protein expression and cell viability were significantly reduced in HMC3 cells treated with 160µM Dox for 12 h.

### Dox Induces JNK/p53-Dependent Apoptosis *via* CALR Protein Inhibition

**Figure 6:**
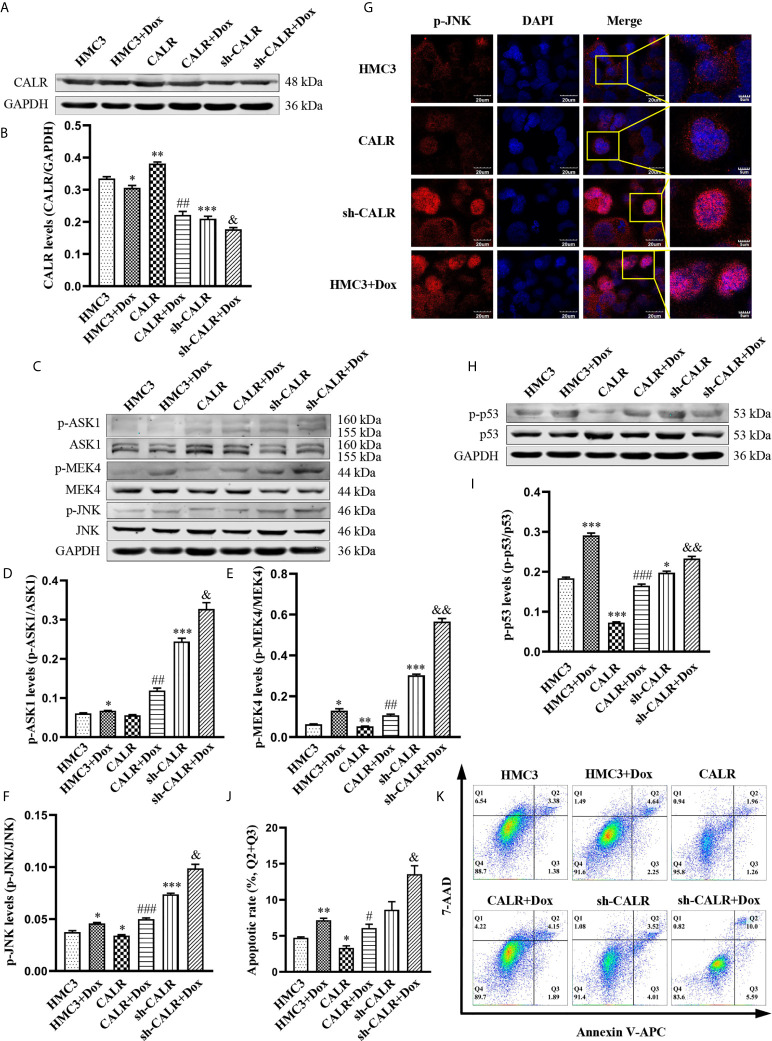
Dox inhibits CALR protein expression and further induces JNK/p53-dependent apoptosis. **(A)** Expression of CALR protein in each group was detected by western blot assay; **(B)** GAPDH was used as an internal reference to calculate CALR relative protein expression. **(C)** Expression levels of p-ASK1, p-MEK4 and p-JNK were determined using western blot; **(D–F)** relative optical density was separately computed by calculating the ratio of p-ASK1, p-MEK4 and p-JNK phosphorylation to total protein. **(G)** Nuclear localization of the p-JNK protein was determined with immunofluorescence microscopy. Scale bars = 20 µm. **(H)** p-p53 protein levels were analysed using western blotting and **(I)** densitometry was use to perform quantitative analysis of p-p53 levels. **(J, K)** HMC3 cell apoptosis was assessed by flow cytometry (Q1: necrotic cells, Q2: late apoptotic cells, Q3: early apoptotic cells, Q4: survival cells) and the rates of apoptosis in the different experimental groups were subjected to statistical analysis, the total apoptosis calculated as (Q2 + Q3). Data represent the means ± SD from three replicates; ^*^ *P* < 0.05, ^**^ *P* < 0.01, ^***^ *P* < 0.001 *versus* HMC3; ^#^ *P* < 0.05, ^##^ *P* < 0.01, ^###^ *P* < 0.001 *versus* CALR; ^&^ *P* < 0.05, ^&&^ *P* < 0.01 *versus* sh-CALR.

To confirm that Dox can activate the JNK/p53 signaling pathway and induce apoptosis in HMC3 cells by inhibiting CALR protein expression, we treated each of the 3 cell lines (HMC3, HMC3-CALR and HMC3-sh-CALR) with 160µM Dox for 12 h. Western blot assays revealed that CALR protein expression was significantly decreased in the Dox-treated groups compared with the corresponding control groups and that CALR protein expression in the HMC3+Dox and sh-CALR groups was reduced relative to the HMC3 group, while that of the CALR group was increased (**Figures 6A, B**). The protein levels of p-ASK1, p-MEK4 and p-JNK were significantly higher in the Dox-treated groups, compared with those in the respective controls. These three proteins were also markedly increased in the HMC3+Dox and sh-CALR groups relative to the HMC3 group, while the protein levels of p-MEK4 and p-JNK were reduced in the CALR group compared with those in the HMC3 group (**Figures 6C–F**). Using immunofluorescence micrsocopy to determine nuclear translocation of p-JNK protein, we also confirmed that the amount of nuclear p-JNK protein in the HMC3+Dox and sh-CALR groups was significantly increased compared with that in the HMC3 group (**Figure 6G**). Western blot analysis also indicated significantly elevated p-p53 protein levels in the Dox-treated groups compared with the corresponding controls; compared with the HMC3 group, the HMC3+Dox and sh-CALR groups demonstrated increased protein levels of p-p53 while the CALR group showed decreased p-p53 protein expression (**Figures 6H, I**). Flow cytometry (**Figure 6K**) showed that the numbers of apoptotic cells in the Dox-treated groups were significantly increased compared with the corresponding controls. And compared with the HMC3 group, the rate of apoptosis was notably increased in the HMC3+Dox group while decreased in the CALR group (**Figure 6J**). These results demonstrate that Dox inhibits CALR protein expression, further activates JNK/p53 signaling pathway, and induces HMC3 cells apoptosis.

### Dox Induces Apoptosis in *B. suis* S2-Infected HMC3 Cells

**Figure 7:**
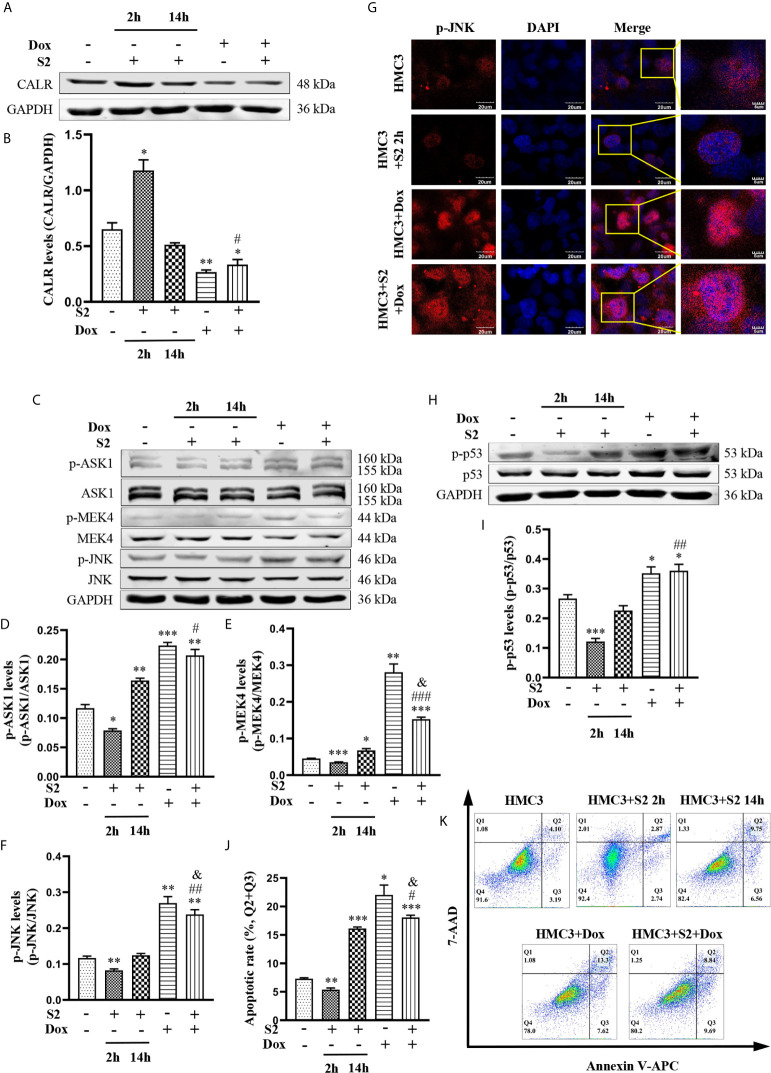
The mechanism of *B. suis* S2-infected HMC3 cells apoptosis induced by Dox. **(A)** CALR protein levels in each group were determined by western blotting and **(B)** relative expression of CALR protein was computed using GAPDH as an internal reference. **(C)** Levels of p-ASK1, p-MEK4 and p-JNK proteins were determined with western blot and **(D–F)** ratios of phosphorylated proteins to total proteinswere evaluated for ASK1, MEK4 and JNK. **(G)** Translocation of p-JNK into nuclei was determined with immunofluorescence microscopy. Scale bars = 20 µm. **(H)** Protein levels of p-p53 were determined by western blot and **(I)** the p-p53 protein bands were quantified using GAPDH as the internal reference protein. **(J)** Apoptosis in HMC3 cells was evaluated by flow cytometry (Q1: necrotic cells, Q2: late apoptotic cells, Q3: early apoptotic cells, Q4: survival cells) **(K)** apoptosis rates are shown, the total apoptosis calculated as (Q2 + Q3). Data are expressed as means ± SD from four independent experiments; ^*^ *P* < 0.05, ^**^ *P* < 0.01, ^***^ *P* < 0.001 *versus* HMC3; ^#^ *P* < 0.05, ^##^ *P* < 0.01, ^###^ *P* < 0.001 *versus* HMC3+S2 14 h; ^&^ *P* < 0.05 *versus* HMC3+Dox.

To further investigate the mechanism of Dox in the induction of apoptosis in *B. suis* S2-infected HMC3 cells, we exposed HMC3 cells to *B. suis* S2 (MOI=50) alone for 2 h or 14 h; treated HMC3 cells with 160µM Dox alone for 12 h; or exposed HMC3 cells to *B. suis* S2 (MOI=50) for 2 h, then treated them with 160µM Dox for 12 h. CALR protein levels and the JNK/p53 signaling pathway-related phosphorylated proteins were evaluated by western blot assay (**Figures 7A, C**). Results showed that the protein levels of CALR in the HMC3+S2+Dox group were significantly lower than in the HMC3 and HMC3+S2 14 h groups (**Figure 7B**), and that, compared with the HMC3 and HMC3+S2 14 h groups, p-ASK1, p-MEK4 and p-JNK protein levels were significantly increased in the HMC3+S2+Dox group (**Figures 7D–F**). However, compared with the HMC3+Dox group, p-MEK4 and p-JNK protein levels in the HMC3+S2+Dox group were decreased significantly (**Figures 7E, F**). Immunofluorescence microscopy showed a significant increase in levels of nuclear p-JNK in the HMC3+S2+Dox and HMC3+Dox groups compared with the HMC3 group (**Figure 7G**). Western blot analysis also demonstrated that the p-p53 protein levels in the HMC3+S2+Dox group were significantly higher than in the HMC3 and HMC3+S2 14 h groups (**Figures 7H, I**). The apoptotic cells were identified by flow cytometry, which indicated that the rate of apoptosis was significantly increased in the HMC3+S2+Dox group compared with that in the HMC3 and HMC3+S2 14 h groups. However, the apoptosis ratio was significantly decreased in the HMC3+S2+Dox group compared with that of the HMC3+Dox group (**Figures 7J, K**). These data suggest that Dox inhibits CALR protein expression, further activates the JNK/p53 signaling pathway, and induces apoptosis in *B. suis* S2-infected HMC3 cells. Furthermore, the data also corroborate that *B. suis* S2 inhibits Dox-induced HMC3 cells apoptosis.

## Discussion

Neurobrucellosis is a common and serious complication of systemic *Brucella* infection (Turkoglu et al., 2018), caused by *Brucella* invasion of the microglia, astrocytes and brain endothelial cells (García Samartino et al., 2010; Marim et al., 2017; Miraglia et al., 2018). Hence, neurobrucellosis pathogenesis warrants further investigation in order to uncover novel therapeutic targets for treatment of neurobrucellosis. In the present study, we found that *B. suis* S2 induced an increase in CALR protein and inhibited apoptosis of HMC3 cells; it also confirmed that CALR protein inhibits apoptosis of HMC3 cells by suppressing phosphorylation of JNK and p53 proteins. Motivated by these findings, we also demonstrated that *B. suis* S2 inhibits the JNK/p53 signaling pathway and prevents apoptosis of HMC3 cells by promoting the expression of CALR protein, while Dox exerts the opposite effect. Finally, we confirmed that Dox activates the JNK/p53 signaling pathway and induces *B. suis* S2-infected HMC3 apoptosis by inhibiting CALR protein expression, and we additionally confirmed that *B. suis* S2 also inhibits the Dox-induced HMC3 cells apoptosis, thus offering promising potential therapeutic targets for neurobrucellosis.

*Brucella* is able to escape the killing action of professional phagocytes by replicating diffusely within the endoplasmic reticulum of macrophages (Ma et al., 2020). As a multifunctional protein in the endoplasmic reticulum, CALR plays a role in anti-apoptosis through regulating calcium ion concentration (Gold et al., 2010). Investigators have previously shown that endothelial cells pretreated with exogenous CALR protein can inhibit expression of apoptosis-associated proteins and reduce apoptosis (Nair et al., 2018). Our results similarly showed that HMC3 cells infected with *B. suis* S2 at an MOI of 50 for 2 h can promote expression of CALR protein and inhibit apoptosis. Since pro-apoptotic signals activate JNK and further phosphorylate p53 to induce apoptosis (Zeke et al., 2016), we also determined protein levels of p-JNK and p-p53 in the *B. suis* S2-infected HMC3 cells. We demonstrated that cells infected with *B. suis* S2 at an MOI of 50 for 2 h significantly inhibited protein levels of p-JNK and p-p53 in HMC3 cells. Therefore, we hypothesize that the inhibitory effect of *B. suis* S2 on the p-JNK and p-p53 protein levels and apoptosis of HMC3 cells may be correlated with the increased expression of CALR protein. Previous research demonstrating that CALR protein can diminish JNK and p53 activation (Zamanian et al., 2016) also supports our hypothesis. In order to test this possibility, we constructed HMC3 cell lines with CALR overexpression and CALR downregulation. Our experiments with these cell lines demonstrated that CALR indeed inhibits protein levels of p-JNK and p-p53 and suppresses apoptosis in HMC3 cells. In conclusion, these results confirm that *B. suis* S2 suppresses protein levels of p-JNK and p-p53 and inhibits HMC3 cells apoptosis through up-regulation of the CALR protein expression.

JNK is a member of the mitogen activated protein kinase (MAPK) family (Schellino et al., 2019). Microbial pathogens can activate ASK1, a member of mitogen-activated protein 3 kinase (MAP3K), which leads to phosphorylation and activation of MKK4 (Ha et al., 2019). The phosphorylated MKK4 synergistically activates JNK *via* phosphorylation of threonine and tyrosine residues (Fleming et al., 2000). To further confirm the inhibition of JNK/p53 signaling pathway by *B. suis* S2, we infected HMC3, HMC3-CALR, and HMC3-sh-CALR cell lines with *B. suis* S2 at an MOI of 50 for 2 h. Our data showed that *B. suis* S2 inhibits the JNK signaling pathway by promoting CALR protein expression. Activated JNK can phosphorylate substrates such as p53, inducing p53-dependent apoptosis (Lien et al., 2015). However, our data showed that *B. suis* S2 lowers p-p53 protein levels to inhibit apoptosis of HMC3 cells by promoting CALR protein expression, which is demonstrated from the opposite side by the literature mentioned above. Taken together, our results confirm that *B. suis* S2 inhibits activation of the JNK/p53 signaling pathway to suppress apoptosis in HMC3 cells by elevating CALR protein expression.

Apoptosis is considered to be a host defense mechanism to reduce survival of pathogenic intracellular bacteria (Mehrbod et al., 2019). Since a variety of intracellular bacteria such as *Brucella* escape attacks of the immune system by replicating within host cells (Ahmed et al., 2016; Khan et al., 2016; Miller et al., 2017), inducing apoptosis of infected cells can restrict bacterial replication and survival (Behar et al., 2011; Ahmed et al., 2016). Dox is a widely used, inexpensive antibiotic (Duivenvoorden et al., 2007) that exerts its antibacterial properties by suppressing bacterial protein synthesis (Luger et al., 2018), making it useful for the treatment of Gram-negative bacterial infections (Luger et al., 2018). In addition to its antibacterial effects, Dox also exhibits pro-apoptotic activity (Pulvino et al., 2015). Several studies have demonstrated that Dox suppresses tumor growth *via* induction of tumor-cell apoptosis (Shieh et al., 2010). Investigating the effects of Dox on expression levels of CALR protein and HMC3 cell viability, we found that treatment with 160µM Dox for 12 h can significantly reduce CALR protein levels in HMC3 cells, with a concomitant decrease in cell viability. Consequently, we hypothesize that Dox inhibits apoptosis in HMC3 cells by suppressing CALR protein expression. Although Dox has been shown to inhibit the viability of a variety of cells such as Sézary Syndrome cells (Alexander-Savino et al., 2016), breast cancer cells (Lokeshwar, 2011) and T-cell lymphoma cells (Forrester et al., 2018) and to induce apoptosis in these cells (Alexander-Savino et al., 2016), the mechanism of Dox-induced apoptosis is poorly known. To test our hypothesis and further explore the potential mechanism of Dox-induced apoptosis induction in HMC3 cells, we treated HMC3, HMC3-CALR and HMC3-sh-CALR cell lines with 160µM Dox for 12 h. We found that the Dox markedly activates the JNK signaling pathway by decreasing protein expression levels of CALR in the HMC3 cells. The activated JNK is transported to the nucleus and phosphorylates pro-apoptotic proteins such as p53 to promote apoptosis (Lin et al., 2019). We also found that Dox promotes transport of p-JNK to the nucleus and further increases p-p53 protein levels to induce apoptosis by suppressing CALR protein expression in HMC3 cells. In summary, our data confirm that the Dox activates the JNK/p53 signaling pathway to induce the apoptosis of HMC3 cells by reducing CALR protein expression.

As recent study has shown that another intracellular bacterium, *Coxiella burnetii*, survives and replicates within host cells by suppressing apoptosis; as in our study, Dox limits *C. burnetii* survival by inducing apoptosis (Hume et al., 2018), but the specific mechanism of this effect is still unclear. *Brucella* is also an intracellular bacterium that inhibits cell apoptosis to survive and proliferate (Scian et al., 2013; Ma et al., 2020). In the present study, we have demonstrated the mechanism of apoptosis inhibition by *B. suis* S2 as well as the mechanism of Dox induced-apoptosis in HMC3 cells. Hypothesizing that Dox may activate the JNK/p53 signaling pathway to induce the apoptosis of *B. suis* S2-infected HMC3 cells, we infected HMC3 cells with *B. suis* S2 (MOI=50) for 2 h and treated them with 160µM Dox for 12 h. Our results confirmed that Dox activates JNK signaling pathway by inhibiting CALR protein expression in *B. suis* S2-infected HMC3 cells. We also examined nuclear transport of the p-JNK protein, p-p53 protein levels and apoptosis of HMC3 cells in each group. The results suggested that the Dox induces p53-dependent apoptosis in the *B. suis* S2-infected HMC3 cells by promoting transport of p-JNK to the nucleus. Moreover, we also found decreases in the levels of p-MEK4, p-JNK and apoptosis in the Dox treated-infected HMC3 cells in comparison with the Dox treated-non infected HMC3 cells, which confirmed the inhibitory effect of *B. suis* S2 on Dox-induced HMC3 cells apoptosis. We considered that this might be due to the infection of *B. suis* S2 decreases the sensitivity of HMC3 cells to the Dox-induced apoptosis. However, its detailed molecular regulation mechanism needs further study.

In conclusion, our study not only demonstrates that *B. suis* S2 inhibits activation of the JNK/p53 signaling pathway to reduce apoptosis in HMC3 cells by inducing CALR protein expression, but also confirms that Dox activates the JNK/p53 signaling pathway to induce apoptosis of *B. suis* S2-infected HMC3 cells by reducing CALR protein levels. In addition, our data suggest that *B. suis* S2 also inhibits the Dox-induced HMC3 cells apoptosis. These findings may contribute to development of novel therapeutic targets for neurobrucellosis and offer the possibility of new approaches for clinical treatment of this disease.

## Data Availability Statement

The original contributions presented in the study are included in the article material. Further inquiries can be directed to the corresponding author.

## Author Contributions

ZW performed the study and wrote the manuscript. YW conducted the study. ZW* supervised the process. HY and JG revised the manuscript. All authors read and censored the final manuscript. Therefore, all authors have performed the study and take responsibility for the integrity and security of the data.

## Funding

This work was supported by the National Natural Science Foundation of China (grant number 31660030 and 81960233) and the Key Research and Development Project of Ningxia Hui Autonomous Region (grant number 2018BFG02017 and 2019BCG01003).

## Conflict of Interest

The authors declare that the research was conducted in the absence of any commercial or financial relationships that could be construed as a potential conflict of interest.
